# Herbal medicine foot bath for the treatment of diabetic peripheral neuropathy: protocol for a randomized, double-blind and controlled trial

**DOI:** 10.1186/s13063-018-2856-4

**Published:** 2018-09-10

**Authors:** Guanjie Fan, Haoyue Huang, Yuping Lin, Guoqing Zheng, Xianyu Tang, Yu Fu, Hua Wei, Ling Zhao, Zhenjie Liu, Mei Wang, Shidong Wang, Qingbo Li, Zhaohui Fang, Yuehong Zhou, Fang Dai, Xiaotang Qiu

**Affiliations:** 1 Department of Endocrinology, Guangdong Provincial Hospital of Chinese Medicine, Guangzhou, China; 2 Department of Neurology, The Second Affiliated Hospital & Yuying Children’s Hospital of Wenzhou Medical University, Wenzhou, China; 3 School of Second Clinical Medicine, Guangzhou University of Chinese Medicine, Guangzhou, China; 4 Department of Endocrinology, The Affiliated Hospital of Liaoning University of Traditional Chinese Medicine, Shenyang, Liaoning China; 5 Department of Endocrinology, Beijing University of Chinese Medicine Affiliated Dongzhimen Hospital, Beijing, China; 6 Department of Geriatric, Luoyang NO.1 Hospital of Traditional Chinese Medicine, Luoyang, China; 7 Department of Endocrinology, The First Affiliated Hospital of Anhui University of Chinese Medicine, Hefei, 230031 China; 8 Department of Endocrinology, Liuyang Hospital of Chinese Medicine, Changsha, China; 9 Department of Endocrinology, The First Affiliated Hospital of Guiyang College of Traditional Chinese Medicine, Guiyang, China; 10 Department of Endocrinology, Hainan Provincial Hospital of Traditional Chinese Medicine, Haikou, China

**Keywords:** Diabetic peripheral neuropathy, Chinese herbal medicine, External application, Randomized controlled trial, Tangbi Waixi decoction, Diabetic foot

## Abstract

**Background:**

As a common complication of diabetes, the incidence of diabetic peripheral neuropathy (DPN) is 60–70% worldwide. DPN is a major risk factor for diabetic foot, which may lead to foot ulceration and even amputation. The treatment of DPN remains challenging. Our preliminary study demonstrated that the external application of Tangbi Waixi (TW) decoction to the lower extremities relieved clinical symptoms and improved nerve conduction velocity in DPN patients. The aim of this study was to verify the efficacy of TW among DPN patients and evaluate the herb mixture’s safety using rigorous methodological designs.

**Methods/design:**

This study is a multicenter, double-blind, randomized controlled trial. A total of 640 DPN patients will be recruited and randomized to receive a foot bath with either the TW decoction or control drug. Participants will be assessed at baseline and 12 and 24 weeks after treatment. The primary outcome was the change of the Toronto Clinical Scoring System (TCSS). Secondary outcomes were nerve conduction velocity, blood glucose, blood lipids, serum inflammatory cytokines, and the European Quality of Life Five-Dimension Scale (EQ-5D) and TCM symptom scores.

**Discussion:**

This multicenter, prospective, randomized clinical trial will provide valuable data regarding the efficacy and safety of foot bath treatment with TW decoction. Positive results would provide a novel treatment regimen for DPN patients.

**Trial registration:**

Chinese Clinical Trial Registry, ChiCTR-IOR-16009331. Registered on 8 October 2016.

**Electronic supplementary material:**

The online version of this article (10.1186/s13063-018-2856-4) contains supplementary material, which is available to authorized users.

## Background

Statistics from the International Diabetes Federation (IDF) demonstrate that there were approximately 425 million people with diabetes worldwide in 2017; it is estimated that the number will increase to 629 million by 2045 [1]. The prevalence of diabetes and pre-diabetes in China has increased to 11.6% and 50.1%, respectively, due to a high calorie intake, sedentary lifestyle, and other risk factors [2, 3]. Diabetic peripheral neuropathy (DPN) is a common chronic complication of diabetes mellitus, with a lifetime prevalence of approximately 50% [4–6]. The clinical symptoms of DPN include itching, numbness, burning sensation, hyperalgesia, and limb weakness, which have a severe negative influence on patient quality of life [7, 8]. DPN is considered a major risk factor for diabetic foot and secondary diseases such as foot ulceration and even amputation [9]. Currently available clinical options for patients with DPN include the following: etiological removal; neurotrophic drugs; and symptomatic treatment; data from several studies did not support positive efficacy [10–12]. Therefore, it is reasonable to develop an effective, safe, and cost-balanced strategy for these patients.

DPN is a microvascular complication caused by local ischemia and neuropathy. Biopsies have revealed graded structural changes in the nerve microvasculature ranging from mild to severe neuropathy [13]. Neuropathy symptoms are more severe in the lower extremities with poor circulation and rich nerve endings. Therefore, therapies meant to improve the microcirculation might alleviate symptoms of DPN. Unfortunately, the frequent failure of oral or intravenous drug administration is due to insufficient plasma concentration in the distal extremities.

Exploring the therapeutic efficacy of local treatment with complementary or alternative medicine is promising. Herbal foot baths have been developed over thousands of years in China. Foot baths not only improve microcirculation [14, 15], they also promote skin permeability to increase the absorption of drugs, which would efficiently increase the efficient drug concentrations at the lower extremities. In addition, traditional Chinese medicine (TCM) considers DPN as collateral retardation due to blood stasis, which would prevent the flow of Qi and blood from reaching the extremities and thus lead to numbness and pain. Therefore, herbs that actively promote the flow of Qi or disperse blood stasis are usually applied to treat DPN; some herbs have demonstrated efficacy for improving the microcirculation [16]. Several studies demonstrated that herbal foot bath therapy is efficacious for the treatment of DPN. However, these studies were not well-designed and did not provide convincing clinical data to support the application of herbal foot baths [17].

Tangbi Waixi (TW) decoction is composed of seven herbal drugs that can activate the flow of Qi and disperse blood stasis. Our preliminary study demonstrated that foot baths with TW decoction promoted local microcirculation, relieved clinical DPN symptoms, and effectively improved the nerve conduction velocity in DPN patients [18]. However, this preliminary study was limited with a small sample size, single center, and non-double-blind design. Therefore, a multicenter, double-blind, randomized, controlled clinical trial is necessary to further determine the efficacy and safety of TW decoction for DPN treatment. We propose that DPN patients treated with TW foot baths will have more positive clinical outcomes than patients receiving the control drug.

## Methods/design

### Study design

**Fig. 1:**
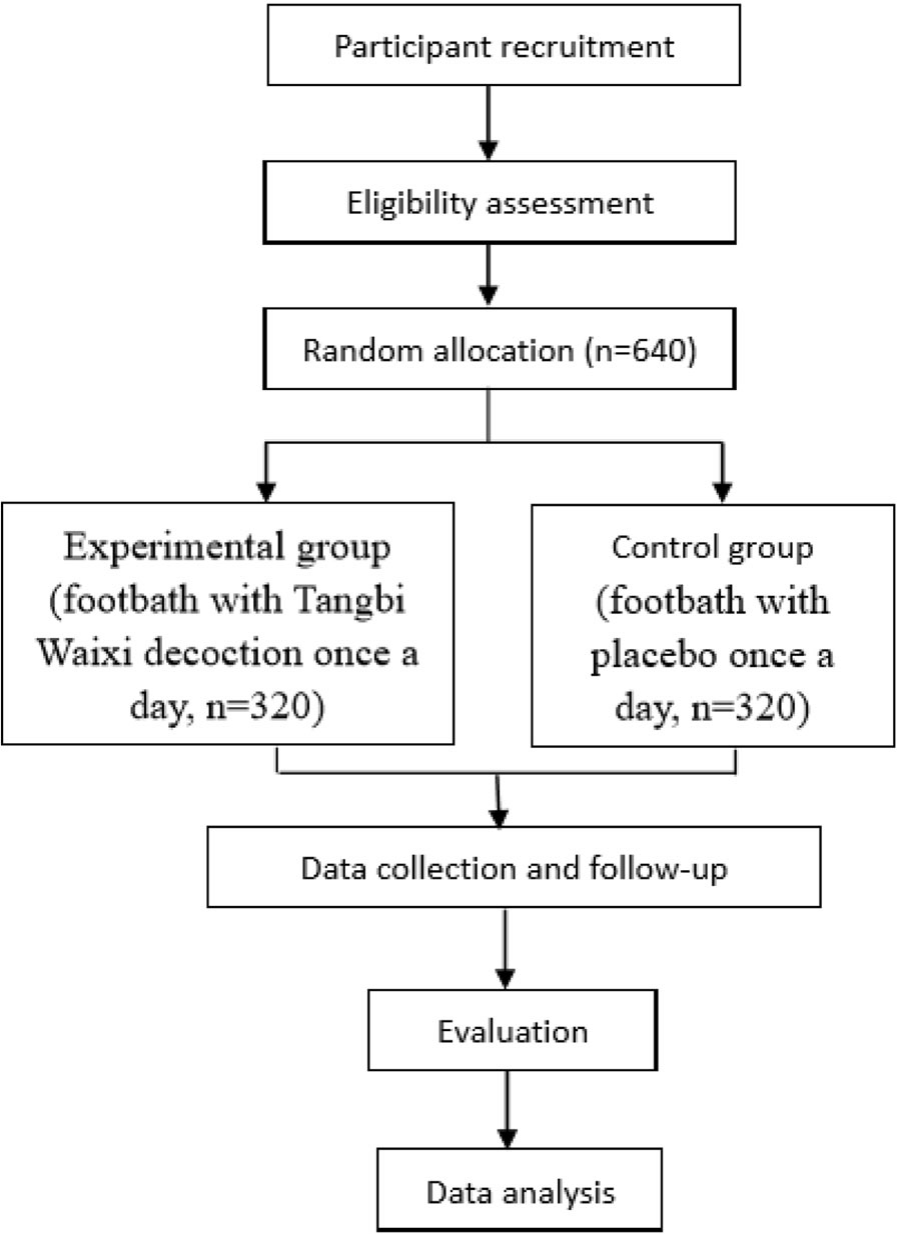
Flow chart of the clinical study

The protocol was designed according to Standard Protocol Items: Recommendations for Interventional Trials (SPIRIT) 2013. For the SPIRIT 2013 Checklist, see Additional file 1. This trial was registered at the Chinese Clinical Trial Registry (ChiCTR-IOR-16009331) and any significant protocol changes were revised accordingly. The prospective trial was designed following the principles of multicenter, double-blinded, and was controlled at a randomization of 1:1. The aim was to evaluate the therapeutic efficacy and safety of TW foot baths for DPN treatment. Patients who agree to participate in the study will be asked to provide written informed consent. Patients will be randomly allocated to either the TW experimental group or the control group after attending an educational session related to the clinical study. Patients will receive a foot bath for half an hour once a day (daytime and night are both acceptable) for two weeks and then stop for two weeks; this cycle constitutes a therapeutic course. All patients will be required to visit their clinicians every two weeks to be evaluated. If their Toronto Clinical Scoring System (TCSS) scores are < 6 points, the foot baths will be terminated and the patients will be followed up. If the TCSS scores are > 6 points, patients will receive a total of three therapeutic courses and then be followed up. All patients will be followed for three months after treatment. During the follow-up period, patients will be required to stop the foot baths and visit their clinicians monthly. Patients who drop out due to side effects or personal reasons will be also followed up. The flow chart of the trial is outlined in Fig. 1.

### Study setting

This multicenter study will be coordinated by Guangdong Provincial Hospital of Chinese Medicine and sponsored by the State Administration of Traditional Chinese Medicine of China. The study will recruit patients from collaborating sites across China, as listed in Table 1. Each research institution will supply similar foot bath equipment that has automatic heating, time setting, and water temperature preservation functions. These conditions will ensure that each patient receives the same foot bath environment at 42 °C lasting for 30 min.

**Table 1:** Collaborating institutions for DPN patient recruitment

◆	Eastern China
	- Beijing University of Chinese Medicine Affiliated Dongzhimen Hospital
	- Affiliated Hospital of Liaoning University of Traditional Chinese Medicine
	- Guangdong Provincial Hospital of Chinese Medicine
	- Hainan Provincial Hospital of Traditional Chinese Medicine
◆	Middle China
	- Anhui Provincial Hospital of Traditional Chinese Medicine
	- Liuyang Hospital of Traditional Chinese Medicine
	- Luoyang No. 1 Hospital of Traditional Chinese Medicine
◆	Western China
	- Guizhou Provincial Hospital of Traditional Chinese Medicine

### Study objectives

The primary objective of this study is to determine the efficacy of TW decoction for improving DPN symptoms, as assessed by TCSS, at the end of treatment and the follow-up period. Secondary objectives include comparison of the side effects experienced in each group over the study period, assessment of nerve conduction velocity between groups at the end of 12 weeks and the follow-up period, and comparison of TCM symptom scores between groups at the end of 12 weeks and the follow-up period. The TCM symptom score will be calculated using the TCM symptoms scoring sheet, which originated from the guiding principles of clinical research on new TCM drugs [19]. Additional secondary objectives will be a comparison of scores calculated by the European Quality of Life Five-Dimension Scale (EQ5D) between groups at the end of 12 weeks and the follow-up period [20, 21] and testing the suitability and feasibility of the proposed outcome measures for the entire clinical trial.

### Eligibility criteria

Patients will be identified and selected by their clinicians based on their TCSS. The inclusion criteria are as follows: men or women aged 18–75 years; a diagnosis of DPN (no limitation on the degree of DPN) [22]; agreement to comply with the clinician’s treatment plan; TCSS scores of ≥ 6 points [23, 24]; and ability to understand and complete the informed consent process and consent to participate in the study.

The exclusion criteria are as follows: other neuropathies such as cervical or lumbar lesions (nerve root compression, spinal stenosis, cervical and lumbar degeneration), cerebral infarction, Guillain-Barre syndrome, or severe arteriovenous vascular lesions (venous thrombosis, lymph tube inflammation); lower extremity skin ulcers or gangrene, lower extremity edema, or suffering from skin diseases; diabetic ketoacidosis, hyperglycemia hypertonic syndrome, diabetes lactic acidosis, and other acute complications; patients with malignant tumors or severe dysfunction of the heart, liver, and kidneys; pregnancy or breast-feeding; participation in other similar interventions; mental disorders that render the patient unable to understand the nature of the study, its scope, and possible outcomes or unable to follow the doctor’s advice.

### Intervention

#### TW decoction and control drug

**Fig. 2:**
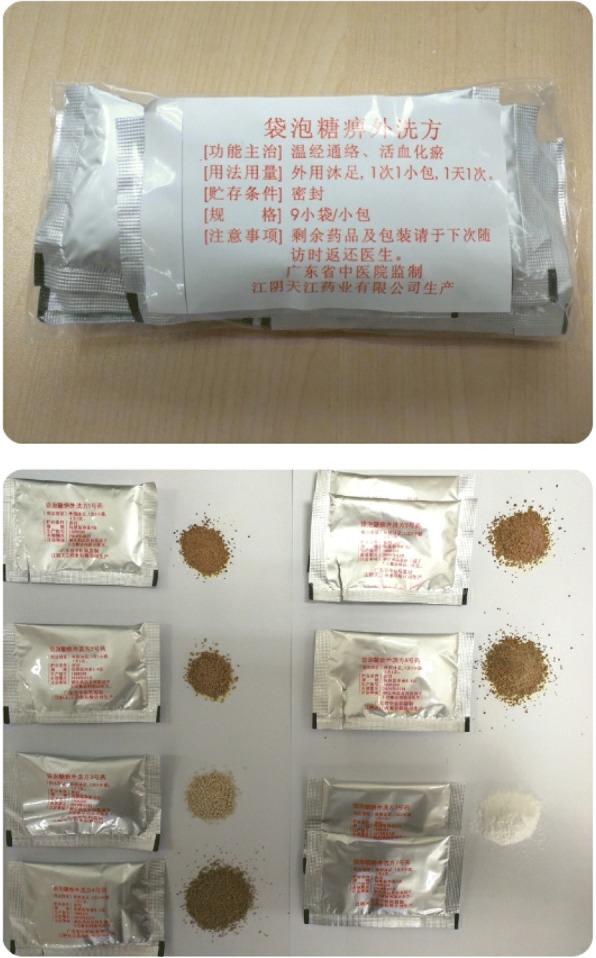
TW decoction or control drug

TW decoction is a Chinese medicinal formula prescribed for DPN treatment. It is composed of cayenne/chili pepper (*Capsicum annuum* Linn.), red Sichuan peppercorns (*Zanthoxylum bungeanum* Maxim.), frankincense (*Boswellia carterii*), myrrh (*Commiphora myrrha*), honeysuckle vine (*Caulis lonicerae*), borneol (derived from the camphor tree, *Cinnamomum camphora*), and safflower (*Carthamus tinctorius*). The control drug will be made of one-tenth of the dry weight of the TW decoction extracts and is similar to the TW decoction in appearance, shape, weight, smell, and color. The herbs for both groups will be extracted and made into granules that are soluble in hot water. Jiangyin Tianjiang Pharmaceutical Company (Wuxi, China) will produce the granules used in this study. TW decoction or control drug is shown in Fig. 2.

The drug will be prepared as follows. The herbs *Capsicum annuum* Linn., *Zanthoxylum bungeanum* Maxim., *Boswellia carterii*, *Commiphora myrrha*, *Caulis lonicerae*, and *Carthamus tinctorius* will be boiled with water separately twice: for 1.5 h the first time and 1 h the second time. The liquid will be filtered, condensed into a paste, and then spray-dried and formed into granules. Borneol will be comminuted into a fine powder. The seven herbal granules will be packed together according to the dose of the TW decoction. As there are seven types of herbal granules that will be packed separately before being combined into one dose, the control drug must be produced separately as well; each herb will have its individual control drug. For the control drug, one-tenth will be composed of the herbal extract and the remaining portion will be composed of lactose, caramel pigment, sunset yellow, lemon yellow, picric acid, etc. to maintain a similar appearance, shape, weight, smell, and color to the corresponding herb.

Patients will be provided with education regarding foot care, diet, and physical activity by an investigator; dose modifications of current antidiabetic, lipid, and blood pressure medications will be allowed at the discretion of the investigator.

### Exclusion of patients receiving certain therapeutic drugs and concomitant treatments

This trial will exclude patients receiving the following therapeutic interventions: neuroprotective drugs; other TCM formulas; and other concomitant treatments. The above events will be discussed with the physician in charge and carefully recorded.

### Outcome measures

**Fig. 3:**
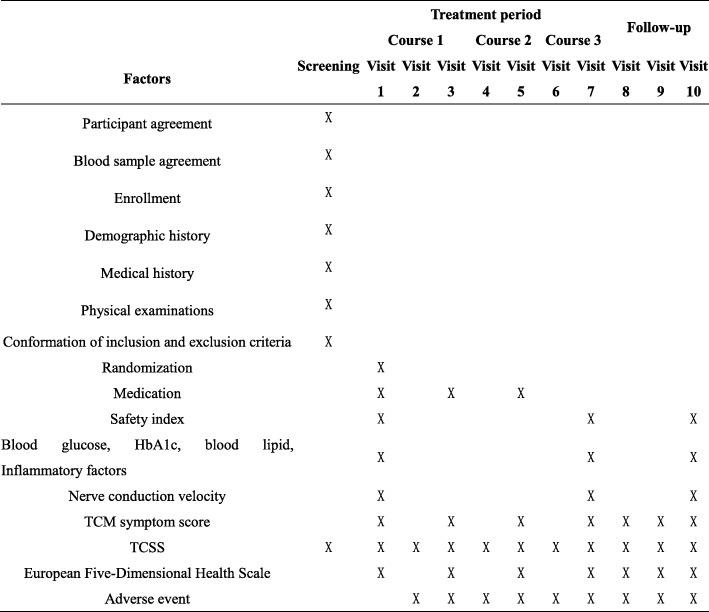
Scheduled events

All of the baseline, in-treatment, and follow-up evaluations will be performed by experienced investigators. All outcome measures are shown in Fig. 3.

### Primary outcome measures

#### Toronto Clinical Scoring System

The TCSS is a scale used to diagnose and evaluate DPN. This scale was proposed by diabetic and neuropathy scholars in Toronto, Canada, in 2001 and they later assessed its validity and reliability [24–26]. In China, researchers also revealed that TCSS has good validity [27].TCSS is composed of three parts: six symptom scores (the presence or absence of foot pain, numbness, tingling, weakness, imbalance, and upper limb symptoms); eight reflex scores (bilateral knee and ankle reflexes); and five physical examination scores (the presence or absence of pinprick, temperature, light touch, vibration, and position sense). Score grading will be stratified as ≤ 5 indicating no neuropathy, 6–8 indicating mild neuropathy, 9–11 indicating moderate neuropathy, and 12–19 indicating severe neuropathy.

### Secondary outcome measures

#### Nerve conduction velocity

All patients will be examined by electromyography at baseline, at the end of the treatment, and at completion of follow-up. The bilateral motor and sensory nerve conduction velocity of the median and common peroneal nerve will be calculated.

#### Blood glucose

Glycemic indices including fasting blood glucose (FBG), postprandial blood glucose (PBG), and glycosylated hemoglobin (HbA1c) will be measured at baseline, at the end of the treatment period, and at completion of follow-up. All blood samples will be taken in the morning: 2 mL fasting venous blood will be collected for measurement of FBG and another 2 mL for HbA1c. For measurement of PBG, 2 mL venous blood will be taken 2 h after breakfast.

#### Blood lipids

Blood lipids indices including total cholesterol (TC), total glycerides (TG), high-density lipoprotein (HDL), and low-density lipoprotein (LDL) will be measured at baseline, at the end of treatment, and at completion of follow-up. Here, 2 mL fasting venous blood will be taken for measurement of blood lipids in the morning.

#### Serum inflammatory cytokines

Serum inflammatory cytokines, including tumor necrosis factor-α (TNF-α), interleukin-6 (IL-6), and C-reactive protein (CRP), will be measured at baseline, at the end of treatment, and at the completion of follow-up. Fasting venous blood will be taken in the morning to measure TNF-α, IL-6, and CRP, each requiring 2 mL blood.

#### European Quality of Life Five-Dimension Scale

EQ5D is a self-administered, easy-to-use questionnaire. It comprises two parts: a self-reported description and a self-rated health status. The self-reported description uses a five-dimensional classification consisting of mobility, self-care, performance level of routine activities, pain/discomfort, and anxiety/depression. Each dimension is graded according to the difficulty in performing the tasks using the following statements: “no problem,” “some problem,” or “extreme problem”. The self-rated health status uses a visual analog scale or “thermometer” (perfect health = 100; worst health = 0) [21, 28]. The validity and reliability of EQ5D have been evaluated by different researchers [20, 29]. The researchers will explain what each dimension means and how to complete the questionnaire before giving it to the participants. Patients are required to complete the questionnaire by themselves after coming to fully understand it. Researchers will collect the questionnaires immediately after patients complete them.

#### TCM symptom score

TCM symptom scores are assessed using a specialized scoring system that is derived from the guiding principles of clinical research on new TCM drugs [19]. The TCM symptom scoring sheet includes four items: limb numbness; limb pain; limb chills; and limb weakness. Each item is divided into four levels and a total of 12 points are calculated (no = 0 points, mild = 1 point, moderate = 2 points, severe = 3 points). Researchers will complete TCM symptom score sheets by interview.

### Patient timelines for study participation

The schedule for enrollment, intervention, assessment, and follow-up are listed in Fig. 3.

### Sample size

The primary outcome measure of the study is TCSS; hence, the sample size will be calculated based on preliminary TCSS results. We anticipate that TCSS scores will decrease 3.5 points in the TW foot bath group compared with 1.5 points in the control group [30, 31]. Based on a two-tailed type-1 error of 0.05 and a type-2 error of 0.099, we anticipate that 266 patients will need to be recruited for each group. Given the possibility of exclusion or lost follow-ups, an increase of 20% will be required for the sample size; therefore, 320 patients will be enrolled in each group.

### Recruitment and consent

Patients that qualify for the study will be identified by their personal physicians and referred to the local research team. The research team consists of clinicians who are familiar with DPN diagnosis and treatment. In addition, the research team will receive appropriate training regarding all the relevant information pertaining to the study. Patients will be screened based on the inclusion and exclusion criteria. If eligible, patients will be informed of the study details and will discuss their health condition and treatment procedures with their case researcher. Researchers at each clinical site will be trained to follow the consent procedures for this study. All patients will be offered a minimum of 24 h to consider enrollment before providing written informed consent. Informed consent will be obtained before the start of the trial.

### Randomization

A block randomization sequence will be generated by SAS 9.2 software (SAS Institute Inc., Cary, NC, USA) with block size set at four, to be performed by an independent staff of the Key Unit of Methodology in Clinical Research (KUMCR) of Guangdong Provincial Hospital of Chinese Medicine. Eligible participants will be randomly allocated to either the experimental group or the control group at a ratio of 1:1 through the Central Randomization Management System developed by KUMCR. At each clinical site, randomization will be also performed at a 1:1 ratio to ensure even distribution.

### Allocation concealment

The randomization list and blinding codes will be kept strictly confidential and only the KUMCR staff will have access to them. Allocation concealment will be ensured until the entire program is complete.

### Blinding

The patients will be blinded to the treatment that they are provided. The investigator and clinical pharmacist will be also blinded to the randomization. Only the KUMCR staff will have access to the randomized treatment allocation table. All investigators, participants, and related medical staff will remain unaware of intervention assignments throughout the trial. The research team will agree to view the blinding allocation only after completion of the trial. In the event of a medical emergency where the patient’s past treatment history is necessary for immediate medical management or treatment decisions, the investigator will un-blind the study. The investigator will attempt to contact the project leader first and then contact the relevant statistician to use a separately arranged emergency code to unlock the blinded treatment code.

### Data collection and management

Data collection will be performed before, during, and after treatment, as well as during the routine follow-up period (Fig. 3). The data will be recorded on the predesigned case report forms (CRF) and entered into an electronic data capture system. All private and sensitive data will be removed. All identification information regarding the patients will be coded.

### Statistical analysis

A statistician blinded to the patient allocation for each group will perform all analyses using PASW Statistics 17.0 software. Statistical analysis will be performed on an intention-to-treat (ITT) basis with 95% CI. The ITT analysis will include all randomized patients. All data will be presented as mean ± standard deviation and compared between groups using unpaired Student’s *t*-tests. Comparisons within a group will be performed using analysis of covariance. Count data will be analyzed using chi-square tests. Repeated measure ANOVA will be performed to assess the changes within and between groups over time. Subgroup analyses will be performed according to the treatment periods. *P* < 0.05 will be considered statistically significant.

### Monitoring

This study will be performed in accordance with the approved protocol. An independent Data and Safety Monitoring Board (DSMB) will be formed before the trial start. The DSMB will review the data after 25%, 50%, and 75% enrollment to monitor the study progress and all adverse events (AEs) that may occur.

### Adverse events

All AEs will be recorded and any serious adverse events (SAEs) will be reported to the Research Ethics Committee (REC) within 24 h. When AEs occur, the study investigators will request that the patient terminate the foot bath and then determine whether this event was related to the investigational formula. The chief investigator may implement urgent safety measures to protect patients from immediate harm. If the AE is related to the investigational formula, it will be termed a side effect. If the side effect is mild and the patient agrees to commence, the foot bath treatment will continue after the symptoms abate. Patients will withdraw from the study if SAEs are observed.

## Discussion

The current clinical trial protocol has been designed as a large, multicenter, double-blind, randomized controlled study to evaluate the efficacy of TCM foot bath intervention for DPN treatment. As a common complication of diabetes, DPN occurs in approximately 60–70% of patients with diabetes mellitus worldwide. Diverse mechanisms are involved in the pathogenesis of DPN [32]. Glucose control plays a significant role in treating DPN, but is not adequate to prevent, arrest, or reverse damaged nerve function [33]. Several studies have demonstrated that pain medications have a beneficial effect on DPN symptoms but are limited by side effects [34]. It is challenging for physicians to implement a highly efficient and safe therapy for DPN [35]. Alternative therapies have been considered for DPN treatment due to their advantages of lower side effects and excellent efficacy. Herbal foot bath is an alternative therapy usually applied for DPN treatment_._ Herbs that promote circulation and eliminate stasis are believed to improve symptoms [36]. Although several studies have reported the efficacy of foot baths for DPN treatment, these studies were limited in scale and were not double-blind [37, 38].^.^ Our proposed clinical study should be the first large, multicenter, double-blind, randomized controlled study to assess the efficacy and safety of foot baths with TCM for DPN treatment.

DPN diagnosis is usually made with “the presence of symptoms and/or signs of peripheral nerve dysfunction in people with diabetes after exclusion from other causes” [39]. It requires the assessment of multiple features of neuropathy because DPN affects a variety of nerve fibers. The TCSS was proposed by diabetic and neuropathy scholars at Toronto, Canada in 2001. It is composed of three parts: a neurological symptom score; a nerve reflex score; and a sensory function examination score [24]. TCSS evaluates the severity of DPN using a scoring system: 6–8 points indicates mild neuropathy; 9–11 indicates moderate neuropathy; and 12–19 indicates severe neuropathy. Compared with other scoring systems, TCSS incorporates both symptoms and signs assessments and could therefore be considered more suitable and accurate for comparative studies.

An appropriate control group is critical for designing a high-quality clinical trial. Jiangyin Tianjiang Pharmaceutical Company was authorized to produce the formulas that are used in this clinical study. The control drug contains one-tenth of the dose that is contained in the TW formula; thus, it is difficult to distinguish the experimental and control drugs based on the color, external packing, or smell. To ensure that the formulas are more convenient to use, the herbs will be extracted and the liquid will be formed into granules that are soluble in hot water.

Another advantage of the proposed study is the use of intelligent thermostatic foot bath apparatuses that will be provided by the same supplier. Because some DPN patients have lost their local thermal sensation, they could easily scald their feet if the water temperature is too high or the foot bath time is too long. This intelligent foot bath device will maintain the water temperature at 42 °C and ensure that the bath lasts for 30 min. To avoid hygiene issues, each patient will be allocated his/her own foot bath device.

### Trial status

Recruitment for the trial started in November 2016 and is expected to end in December 2018.

## Additional file

Additional file 1: Spirit checklist. (DOCX 30 kb)

## Abbreviations

CRP: C-reactive protein
DPN: Diabetic peripheral neuropathy
DSMB: Data and Safety Monitoring Board
EQ5D: European Quality of Life Five-Dimension Scale
FBG: Fasting blood glucose
HDL: High-density lipoprotein
IDF: International Diabetes Federation
IL-6: Interleukin-6
LDL: Low-density lipoprotein
PBG: Postprandial blood glucose
REC: Research Ethics Committee
SAEs: Serious adverse events
TC: Total cholesterol
TCM: Traditional Chinese medicine
TCSS: Toronto Clinical Scoring System
TG: Total glyceride
TNF-α: Tumor necrosis factor-α

## References

[1] International Diabetes Federation (2017). IDF diabetes atlas eighth edition 2017.

[2] Xu Y, Wang L, He J (2013). Prevalence and control of diabetes in Chinese adults. JAMA.

[3] Yang W, Lu J, Weng J (2010). Prevalence of diabetes among men and women in China. N Engl J Med.

[4] Juster-Switlyk K, Smith AG (2016). Updates in diabetic peripheral neuropathy. F1000Res.

[5] Mehra M, Merchant S, Gupta S (2014). Diabetic peripheral neuropathy: resource utilization and burden of illness. J Med Econ.

[6] Tesfaye S, Selvarajah D (2012). Advances in the epidemiology, pathogenesis and management of diabetic peripheral neuropath. Diabetes Metab Res Rev.

[7] Weintraub MI, Wolfe GI, Barohn RA (2003). Static magnetic field therapy for symptomatic diabetic neuropathy: a randomized, double-blind, placebo-controlled trial. Arch Phys Med Rehabil.

[8] Jensen MP, Friedman M, Bonzo D (2006). The validity of the neuropathic pain scale for assessing diabetic neuropathic pain in a clinical trial. Clin J Pain.

[9] Amin N, Doupis J (2016). Diabetic foot disease: from the evaluation of the “foot at risk” to the novel diabetic ulcer treatment modalities. World J Diabetes.

[10] Kim H, Kim JJ, Yoon YS (2012). Emerging therapy for diabetic neuropathy: cell therapy targeting vessels and nerves. Endocr Metab Immune Disord Drug Targets.

[11] Zhou JY, Zhang Z, Qian GS (2016). Mesenchymal stem cells to treat diabetic neuropathy: a long and strenuous way from bench to the clinic. Cell Death Discov.

[12] Mahmood D, Singh BK, Akhtar M (2009). Diabetic neuropathy: therapies on the horizon. J Pharm Pharmacol.

[13] Cameron NE, Eaton SEM, Cotter MA (2001). Vascular factors in diabetic neuropathy. Diabetologia.

[14] Matsumoto S, Shimodozono M, Etoh S (2010). Beneficial effects of foot bath in controlling spasticity after stroke. Int J Biometeorol.

[15] Jeong HL, Eun KS, Jae SS (2017). The effects of aroma massage and foot bath on psychophysiological response in stroke patients. J Phys Ther Sci.

[16] Xu WG (2007). Sixty-one patients with diabetic peripheral neuropathy treated by Tongluo Yangyin recipe. Chin J Integr Med..

[17] Xiong GH, Li Y (2015). Research situation of treating diabetic peripheral neuropathy with traditional Chinese medicine leg and foot bathing therapy. Glob Tradit Chin Med.

[18] Fan GJ, Tang XY, Liu ZJ (2011). Herbal bath therapy combined with Mecobalamin on diabetic peripheral neuropathy clinical observation. New J Tradit Chin Med.

[19] Zheng XY (2002). Guiding principles of clinical research on new drugs of TCM.

[20] Brooks R (1996). Euro Qol: the current state of play. Health Policy.

[21] Wang H, Kindig DA, Mullahy J (2005). Variation in Chinese population health related quality of life: results from a Euro Qol study in Beijing. China Qual Life Res.

[22] Sima AA, Thomas PK, Ishii D (1997). Diabetic neuropathies. Diabetologia.

[23] Lou DJ, Zhu QQ, Si XW (2013). Application of Toronto clinical scoring system in screening diabetic peripheral neuropathy in T2DM patients. Chin J Diabetes.

[24] Perkins BA, Olaleye D, Zinman B (2001). Simple screening tests for peripheral neuropathy in the diabetes clinic. Diabetes Care.

[25] Bril V, Perkins BA (2002). Validation of the Toronto clinical scoring system for diabetic polyneuropathy. Diabetes Care.

[26] Bril V, Tomioka S, Buchanan RA (2009). Reliability and validity of the modified Toronto Clinical Neuropathy Score in diabetic sensorimotor polyneuropathy. Diabet Med.

[27] Liu F, Mao JP, Yan X (2008). Toronto clinical scoring system in diabetic peripheral neuropathy. Zhong Nan Da Xue Xue Bao Yi Xue Ban.

[28] Dhillon V, Hurst N, Hannan J (2005). Association of low general health status, measured prospectively by Euroqol EQ5D, with osteoporosis, independent of a history of prior fracture. Osteoporos Int.

[29] Xing YB, Ma AX (2013). Study on reliability and validity of Chinese version of EQ-5D-5L. Shang Hai Yi Yao.

[30] Fan GJ, Tang XY, Liu ZJ (2010). Clinical observation of washing traditional Chinese medicine treatment and mecobalamin on diabetic peripheral neuropathy. J New Chin Med.

[31] Luo GB, Fan GJ, He J (2011). Evaluation of clinical pathway in the treatment of diabetic peripheral neuropathy. J New Chin Med.

[32] Yagihashi S, Mizukami H, Sugimoto K (2011). Mechanism of diabetic neuropathy: where are we now and where to go. Diabetes Investig.

[33] Valk GD, Kappelle AC, Tjon ATAM (1996). Treatment of diabetic polyneuropathy with the neurotrophic peptide ORG 2766. J Neurol.

[34] Çakici N, Fakkel TM, van Neck JW (2016). Systematic review of treatments for diabetic peripheral neuropathy. Diabet Med.

[35] Lundborg GN, Bjorkman AC, Rosen BN (2010). Cutaneous anaesthesia of the lower leg can improve sensibility in the diabetic foot. A double-blind, randomized clinical trial. Diabet Med.

[36] Qu L, Zhang H, Gu B (2016). Jinmaitong alleviates the diabetic peripheral neuropathy by inducing autophagy. Chin J Integr Med.

[37] Hong B, Chen LJ, Ying XC (2015). Clinical observation of Chinese medicine foot bath combined with Alprostadil injection in treating diabetic peripheral neuropathy and effect on serum adiponectin, IL-6 and TNF-α levels. Chin Arch Tradit Chin Med.

[38] Zhang L, Wang XM, Wang GH (2015). Effectiveness of Traditional Chinese Medicine Fumigation on Diabetic Peripheral Neuropathy: A Systematic Review. J Nurs (China).

[39] Boulton AJM, Gries FA, Jervell JA (1998). Guidelines for the diagnosis and out-patient management of diabetic peripheral neuropathy. Diabet Med.

